# CAP: Commutative algebra prediction of protein-nucleic acid binding affinities

**DOI:** 10.1088/2632-2153/ae29bc

**Published:** 2025-12-17

**Authors:** Mushal Zia, Faisal Suwayyid, Yuta Hozumi, JunJie Wee, Hongsong Feng, Guo-Wei Wei

**Affiliations:** 1 Department of Mathematics, Michigan State University, East Lansing, MI 48824, United States of America; 2 Department of Mathematics, King Fahd University of Petroleum and Minerals, Dhahran 31261, Saudi Arabia; 3 Department of Mathematics and Statistics, University of North Carolina at Charlotte, Charlotte, NC 28223, United States of America; 4 Department of Electrical and Computer Engineering, Michigan State University, East Lansing, MI 48824, United States of America; 5 Department of Biochemistry and Molecular Biology, Michigan State University, East Lansing, MI 48824, United States of America; 6 Current address: School of Mathematics, Georgia Institute of Technology, Atlanta, GA, United States of America.

**Keywords:** persistent commutative algebra, facet persistence barcodes, persistent ideals, machine learning, protein-nucleic acid binding

## Abstract

An accurate prediction of protein-nucleic acid binding affinity is vital for deciphering genomic processes, yet existing approaches often struggle in reconciling high accuracy with interpretability and computational efficiency. In this study, we introduce commutative algebra prediction (CAP) framework, which couples persistent Stanley–Reisner theory with advanced sequence embedding for predicting protein-nucleic acid binding affinities. CAP encodes proteins through transformer-learned embeddings that retain long-range evolutionary context, and represents DNA and RNA with *k*-mer algebra embeddings derived from persistent facet ideals, which capture fine-scale nucleotide geometry. We demonstrate that CAP surpasses the SVSBI protein-nucleic acid benchmark and, in a further test, maintains reasonable performance on newly curated protein-RNA and protein-nucleic acid datasets. Leveraging only primary sequences, CAP generalizes to any protein-nucleic acid pair with minimal preprocessing, enabling genome-scale analyses without 3D structural data and promising faster virtual screening for drug discovery and protein engineering.

## 1. Introduction

Protein-nucleic acid recognition underpins a spectrum of essential biological processes. These processes form the backbone of cellular processes central to life, ranging from DNA replication, transcription, genome stability to RNA transport, splicing, and post-transcriptional regulation. Accurate modeling of protein-DNA and protein-RNA binding is crucial not only for fundamental biology but also for therapeutic innovations, accelerating genomic medicine, synthetic biology, and drug design [1]. Molecular-level mechanisms are mediated by proteins through recognition and binding to specific DNA or RNA sequences. Such mechanisms include hydrogen bonding, electrostatic attraction, hydrophobic interaction, physicochemical force, and structural complementarity. Disruption in such binding events can lead to the development of various diseases such as cancer, neurodegenerative conditions, and autoimmune disorders [2]. Thus, the orchestration of these associations is crucial not only for decoding biomolecular functions but also for paving the way to new therapies and drug discovery.

A variety of conventional techniques have been adopted in the past for the gold-standard measurement of binding affinity predictions of protein-protein/ligand systems, like fluorescence spectroscopy, electrophoretic mobility shift assays, surface plasmon resonance, isothermal titration calorimetry, and filter binding [3]. However, they are typically resource-intensive and time-consuming. To address these limitations, a spectrum of in-silico strategies have been developed. Classical physics-based methods, such as thermodynamic integration, free energy perturbation, molecular mechanics, and Poisson–Boltzmann surface area, offer detailed estimates but are particularly laborious for large biomolecular systems. Non-classical approaches include, but are not limited to knowledge-driven potentials [4], empirical scoring functions [5], force-field scoring functions [6], and machine-learning approaches using engineered descriptors [7]. These methods have delivered promising results in protein-protein or protein-ligand systems. However, they tend to underperform on DNA owing to its unique conformational complexities and lack of availability of comprehensive binding affinity datasets.

On the other hand, various experimental and computational approaches have been applied over the past decade to understand protein-RNA interactions, ranging from high-throughput CLIP-seq [8], purely seq-based prediction algorithms [9–11] to knowledge-based scoring [12] and coarse-grained docking [13]. In addition, resolving protein-RNA complex structures through x-ray crystallography or nuclear magnetic resonance is hampered by inherent flexibility of many RNA partners which makes such determinations technically challenging and prohibitively slow. Although several protein-RNA docking approaches have substantially accelerated the discovery of RNA-protein interaction sites and expanded available structural decoys, yet none provide quantitative binding affinity measurements, leaving a paucity of RNA affinity data [14].

In light of these limitations, data-driven models, particularly those built on machine learning (ML) frameworks, have recently reshaped computational molecular biology by emerging as an integral component for modern drug design [15, 16]. The integration of bioinformatics [17] with ML and deep learning, when applied to protein-nucleic acid systems, has demonstrated promising shift by enabling the prediction of molecular interactions in complex high-dimensional data at an unprecedented scale. Recently, mathematical artificial intelligence (AI), particularly topological deep learning first introduced in 2017 [18], has emerged as a new paradigm for rational learning in data science [19], leading to a new frontier for modeling intricate biomolecular systems [20], such as proteins and their interactions [21, 22]. Notably, mathematical AI secured victories in Drug Design Data Resource (D3R) Grand Challenge, an annual worldwide competition series in computer-aided drug design [23, 24]. Recently, topological sequence analysis (TSA) has been proposed as a viable approach for biological sequence modeling [25]. Delta complex approaches of TSA enable the efficient treatment of large genomic sequences [26], while category theory approaches of TSA achieve higher accuracy for nearly identical genetic variants [27].

Some early studies laid important groundwork for protein-DNA binding interactions using ML frameworks, such as Zhao *et al*’s atomic pairwise statistical potential (DDNA3) [28], Rastogi *et al*’s sequencing-based affinity profiling [29], and Barissi *et al*’s physics-based machine-learning method (DNAffinity) [30]. Building on these advances, subsequent models such as PreDBA [31], PDA-Pred [32], and emPDBA [33] have broadened the affinity-prediction toolbox. Likewise, for protein-RNA interactions, Yang *et al* [34] assembled the first quantitative dataset of protein-RNA affinities, which was later followed by the development of several structure-driven learning frameworks, each developed with its own curated dataset, including the methods proposed by Nithin *et al* [5] and PredPRBA [35], as well as models including Hong *et al* [36] and the more recent PRA-Pred [37]. Though these advancements have pushed the field forward, yet most approaches rely on stacked regressors, class-specific framework architectures to curb overfitting in small datasets, and basic interface metrics.

Commutative algebra is the mathematical study of commutative rings, their ideals, modules, and related structures [38]. As a foundational framework in pure mathematical fields such as algebraic number theory, homological algebra, and algebraic geometry, commutative algebra has seen limited application in data science and the biological sciences. In a recent effort, Suwayyid and Wei introduced persistent Stanley–Reisner theory (PSRT) to forge a connection between commutative algebra, algebraic topology, data science, and ML [39]. This method has already yielded promising results in protein-ligand binding affinity predictions via a structure-based approach [40].

The Stanley–Reisner (SR) theory investigates the commutative algebra of simplicial complexes, assemblies of vertices, edges, triangles, and their higher-order faces, through the study of square-free monomial ideals in polynomial rings [41, 42]. PSRT integrates these notions with multiscale analysis, enabling commutative algebra learning and predictions. PSRT examines the evolution of SR ideal across a filtration, yielding a range of algebraic and topological invariants, such as persistent graded Betti numbers (computed via Hochster’s formula), persistent *f* - and *h*-vectors, and persistent facet ideals. In particular, facet-persistence barcodes which document the ‘birth’ and ‘death’ of each facet ideal during filtration have been developed for real-world data applications. Thus, PSRT is a new multiscale approach that offers rich geometric, topological, and combinatorial analysis not readily accessible to conventional mathematical, statistical, and physical approaches.

In this study, we investigate commutative algebra prediction (CAP) framework for the sequence-based modeling of protein-nucleic acid binding affinities, a direction not previously explored. Our CAP offers algebraic sequence analysis, an algebra-based sequence-driven alternative to conventional structure-based approaches, facilitating an effective modeling of biomolecular interactions using primary sequence information alone. We employ a dataset assembled by Shen *et al* [11], comprising 186 protein-nucleic acid complexes derived from the PDBbind v2020 database, referred to here as S186. Each complex in this dataset is represented by pairing rich residue-level embeddings of the protein sequence with nucleotide-level descriptors of the associated nucleic acid strand (DNA or RNA). These complementary sequence-based features implicitly capture interactions such as hydrogen bonding, electrostatic contacts, base stacking, and van der Waals forces. Moreover, it is well recognized that binding alters the electrostatic environment of a complex; in particular, changes in pKa values and protonation states contribute to protein-nucleic acid recognition [43]. CAP reflects these effects implicitly through sequence-derived representations: the ESM2 embeddings encode residue-level biochemical context (charge, polarity, protonation tendencies), while the facet-based nucleic-acid descriptors capture complementary sequence motifs. We evaluate CAP on two newly curated datasets: a protein-RNA dataset (S142) containing 142 complexes, and a protein-nucleic acid dataset (S322) containing 322 complexes. The results demonstrate CAP’s robust predictive performance, highlighting a promising new approach for affinity prediction without explicit reliance on structural models.

In the following sections, we present our results followed by a brief discussion on existing methodologies, the two new datasets, and feature extraction processes with a detailed demonstration of robust predictive performance of CAP for protein-nucleic acid binding affinity. We also elaborate on the mathematical foundations and computational interpretability of CAP. Finally, we conclude by discussing our findings and outlining potential future research directions.

## 2. Results and discussion

### 2.1. Overview of the CAP model

**Figure 1.**
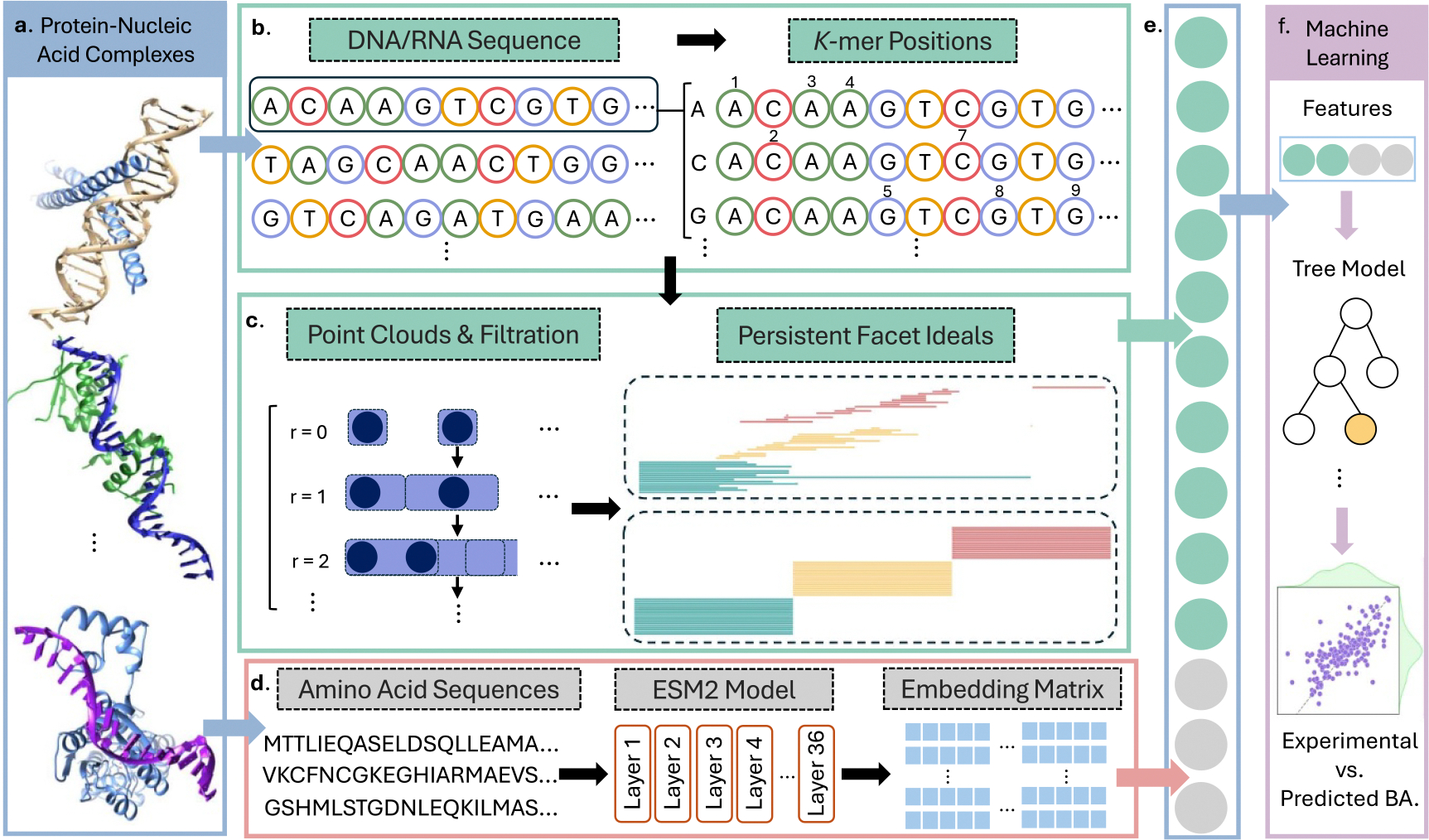
Illustration of the workflow in CAP. (a) Protein-nucleic acid complexes. (b) *k*-mers are extracted from the DNA sequences of the given complexes. For each *k*-mer = 1, the set of its occurrence positions within the sequence is treated as an input data. (c) The persistent facet ideals associated with these input data for three dimensions are then computed and used as topological features for the corresponding *k*-mers. (d) From the amino acid sequence for the given complexes, 2560 embedding vectors are generated using state-of-the-art ESM2 model with 36 layers. (e) The feature vectors of all DNA and proteins are concatenated to construct a genome-level topological representation. (f) Finally, our CAP model is trained using these features with machine learning techniques for the prediction task.

Figure 1 illustrates the workflow of the proposed CAP model. For a given protein-nucleic acid complex (a), the nucleic acid sequence is processed with PSRT (b–c) to provide a CAP feature vector. Meanwhile, the protein sequence is processed with the ESM2 model to result in an embedding matrix (d). The combined feature vector (e) is fed into an ML model (f). More details of protein sequence embedding, nucleic acid sequence analysis, and ML parametrization can be found in the methods section.

### 2.2. Protein-nucleic acid binding affinity prediction

A vital category of biomolecular interactions involves protein-nucleic acid binding which underpins essential cellular processes such as catalysis, molecular transport, signal transduction, transcription, and translation. Moreover, these interactions preserve chromosomal integrity and regulate gene expression; however, they can contribute to pathologies including cancer, genetic disorders, and autoimmune diseases. Understanding how hydrogen bonds, van der Waals contacts, dipole-dipole interactions, electrostatics, van der Waals attractions, and hydrophobicity contribute to binding affinities then informs the rational design of new therapeutics such as structure-based drug design. In this study, we apply our CAP framework to capture these interactions and predict protein-nucleic acid binding affinity.

We begin by evaluating the predictive accuracy of our PSRT-guided framework on the S186 dataset against the sequence-based virtual screening of biomolecular interactions (SVSBI) model on protein-nucleic acid binding affinity [11]. Due to lack of established benchmarks, Shen *et al* [11] assembled a dataset of 186 protein-nucleic acid complexes. In order to construct a high-quality dataset from PDBbind-v2020, the authors applied four stringent filters: (i) a complex was retained only if it contained one unique protein sequence and one unique nucleic acid sequence. Furthermore, multiple chains carrying the same sequence were allowed, but any chain with mixed or hybrid bases (e.g. both T and U) was discarded; (ii) only complexes with experimentally determined binding affinities measured at 298K were kept; (iii) entries containing ambiguous labels (~, <, or >) were not considered; (iv) exclude those complexes that whose nucleic acid sequence length is fewer than 5.

**Figure 2.**
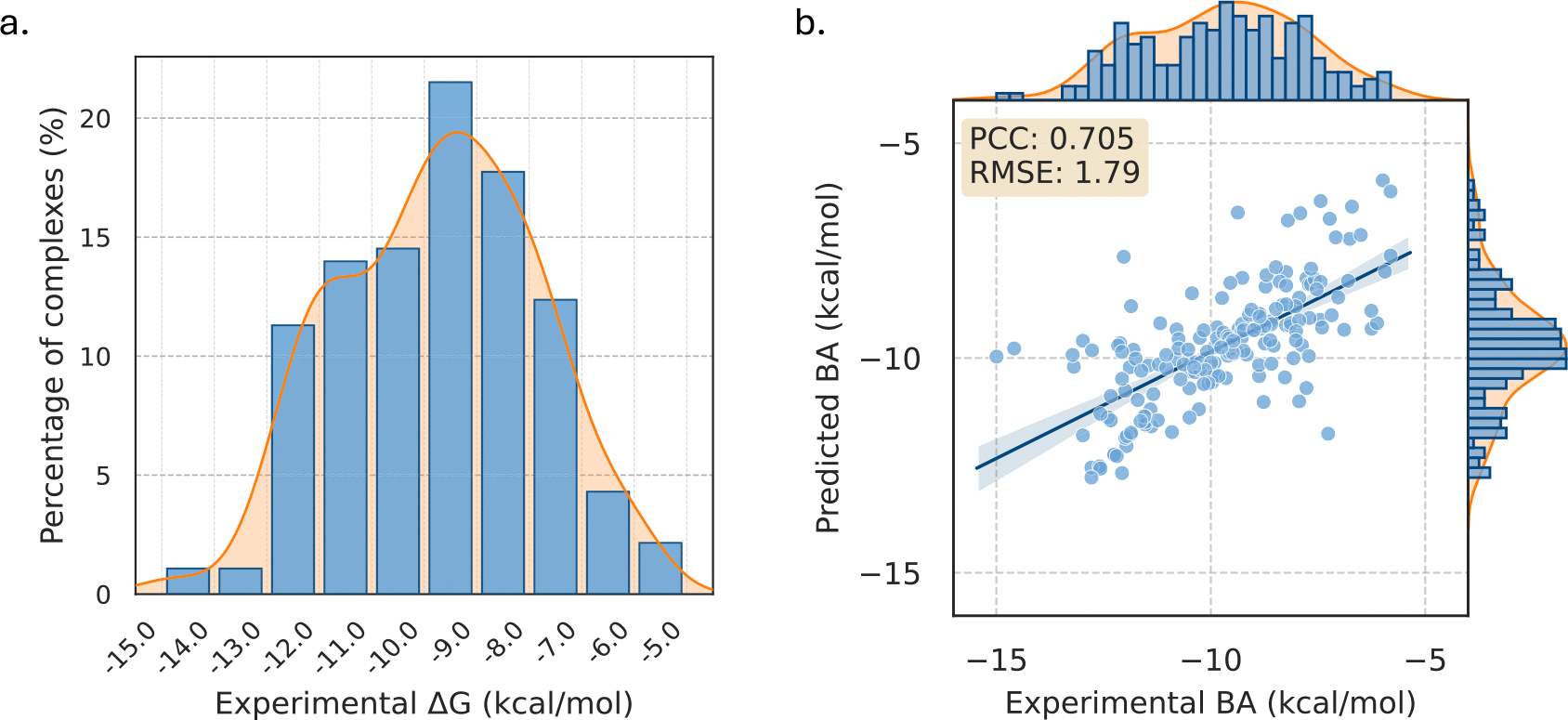
(a) Distribution of experimental binding affinity (ΔG) in the S186 dataset. (b) A comparison between the experimental binding affinities and the predicted values from our CAP model for this dataset. The corresponding Pearson correlation coefficient (PCC) value is 0.705 with an RMSE of 1.79 kcal mol^−1^.

**Figure 3.**
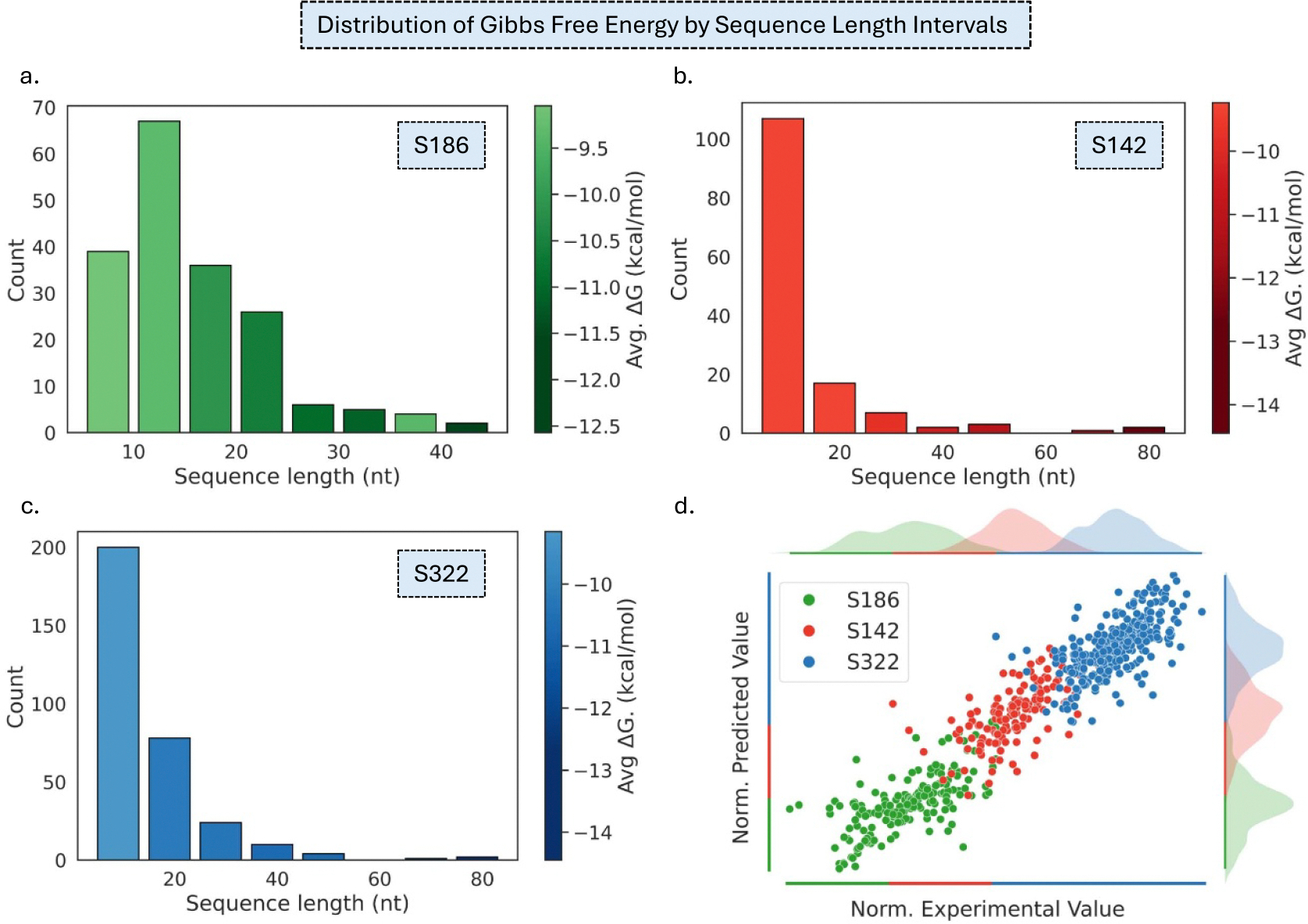
Distribution of experimental Gibbs free energy (ΔG) across sequence length intervals for the datasets (a) S186, (b) S142, and (c) S322. Color intensity encodes mean ΔG; darker shades indicate stronger while lighter shades represents weaker binding. (d) A comparison of normalized experimental and predicted binding affinities of S186, S142, and S322 datasets. Each dataset is scaled to a specific region with an equal range for clear visual illustration.

The SVSBI framework leverages an ESM-based Transformer for proteins and DNABERT for nucleic acids and records an average Pearson of 0.669 and root mean squared error (RMSE) of 1.98 kcal mol^−1^. On the other hand, CAP achieves robust prediction performance with a Pearson correlation coefficient (*PCC*) of 0.705 with RMSE of 1.79 kcal mol^−1^, surpassing the existing SVSBI model [11] for the protein-nucleic binding affinity prediction on the same dataset as shown in table 1. Furthermore, we analyze the distribution of experimental binding affinity ΔG values of S186 as shown in figure 2(a). We also observe that ΔG ranges from −15 kcal mol^−1^ to −6 kcal mol^−1^ and 66% of the complexes have ΔG of −11 to −7 kcal mol^−1^. An illustrative comparison between the experimental binding affinities and the predicted values from our CAP model for this dataset is presented in figure 2(b) and a full comparison of experimental and predicted binding affinities is provided in table S1. In addition, a representation of how binding free energy varies with sequence length in S186 is represented in figure 3(a). While the strand lengths range from 5 to 45ñt, we observe that the shortest strands (5ñt) bind weakly on average (mean $\Delta G\;=\;-7.77\;kcal\;{mol}^{-1}$), whereas the longest strands (45ñt) reach an average $\Delta G\;=\;-9.57\;kcal\;{mol}^{-1}$. Moreover, we also note that the length interval with the highest coverage (10–15ñt, 67 complexes) has a mean ΔG of −9.32 kcal mol^−1^. Our CAP framework provides a powerful and structure-free method, even though that the size of our dataset is modest.

Several existing protein-DNA affinity models [28, 31–33] and protein-RNA models [35–37] have broadened the binding prediction toolbox. However, they depend on large, handcrafted feature sets and category-specific tuning steps that complicate workflows and have limited generalizability. CAP sidesteps these limitations by coupling PSRT with a concise sequence-level interaction embedding and a single gradient-boosting regressor. By summarizing residue-nucleotide contacts directly from the paired protein and nucleic-acid sequences, CAP removes the need to count hydrogen bonds, tally base-pair frequencies, model stacking ensembles, or train separate predictors for different structural subclasses. Because CAP relies solely on primary sequences, it can be applied to any protein-DNA/RNA pair without 3D structures, subclass labels, or bespoke feature engineering, offering higher predictive power with far less manual effort and providing an extensible framework for large-scale studies of protein-nucleic recognition.

## 2.3. Discussion

Within the spectrum of existing binding affinity approaches for protein-DNA, the most recent is emPDBA [33], which integrates 106 sequence- and structure-based features into a stacking ensemble applied to 340 protein-DNA complexes at 40% protein sequence similarity. After subclassifying DNA by structural form and applying feature selection method, it reaches a correlation value of 0.66 with mean absolute error (MAE) = 1.24 kcal mol^−1^, compared with 0.12 (MAE = 1.64 kcal mol^−1^) on an unclassified set. Another model, PDA-Pred [32], built on 117 features for each of the 391 complexes at 25% sequence identity, employs similar classifications of DNA structures and with an additional protein functional categorization followed by a feature selection method (jack-knife) to achieve *PCC* = 0.78, dropping to just *PCC* = 0.21 without any categorization. Furthermore, on a common independent testing set of 36 complexes, emPDBA showed an *PCC* = 0.53 with MAE = 1.11 kcal mol^−1^, whereas earlier ensemble regression methods like PreDBA [31] attained *PCC* = 0.30, and the statistical potential DDNA3 [28] only *PCC* = 0.09 with MAE = 2.05 kcal mol^−1^ and MAE = 1.80 kcal mol^−1^, respectively.

While numerous models exist for protein-DNA binding affinity prediction, there are only a limited number of models available for predicting protein-RNA binding affinity [35–37]. The most recent, PRA-Pred model [37], consists of 217 protein-RNA complexes with proteins clustered at a 25% sequence identity cutoff, adopts a classification strategy similar to that used in protein-DNA binding affinity prediction. It categorizes RNA into five structural subclasses and proteins into functional categories and achieves a Pearson correlation of 0.77 and a MAE of 1.02 kcal mol^−1^ using a jack-knife feature selection strategy. On the standard blind set of 44 complexes, the model obtains an overall performance of *PCC* =0.60 with an MAE of 1.47 kcal mol^−1^. Another model, PRdeltaGPred [36], starts with 63 interface-based features and applies a custom feature reduction process to improve prediction accuracy. Despite this tailored feature selection strategy, PRdeltaGPred reports *PCC* =0.41 with an MAE of 1.83 kcal mol^−1^ on the standard 44-complex blind set. The third model, PredPRBA [35], is trained on 103 protein-RNA complexes filtered at a 40% sequence identity cutoff. It makes use of both sequence-level and structural features at the whole-complex level, and applies jack-knife test within six predefined RNA subclasses. While the model’s overall performance on the standard 44-complex blind test is limited (*PCC* =0.07, MAE = 2.07 kcal mol^−1^, its best pre-classification performance reaches *PCC* =0.48, suggesting that feature and label heterogeneity across RNA types poses a challenge for unified modeling.

These earlier studies on protein-DNA/RNA affinity predictions have reported results on datasets clustered anywhere from strict 25% identity to no clustering at all, making headline metrics hard to compare across papers. In our study, we employ the S186 dataset [11] that solely focus on biochemical uniformity (single protein-nucleic complex per entry, unambiguous labels, identical assay temperature). The resulting dataset, though modest in size, spans a balanced range of binding energies and local neighborhoods, so model performance is driven by genuine physico-chemical signal rather than by the occasional near-duplicate chain. Encouraged by these stable results and to further assess our CAP framework on protein-RNA binding affinity, we curated the S142 dataset of 142 protein-RNA complexes. On this set, our model achieves a *PCC* of 0.653 and an RMSE of 2.18 kcal mol^−1^; evaluation on an expanded dataset, S322, of 322 protein-nucleic acid complexes yields better performance (*PCC* = 0.669, RMSE = 2.0 kcal mol^−1^). A prediction performance of both datasets can be found in table 2, whereas a full comparison of experimental and predicted binding affinities for S142 and S322 are presented in tables S2 and S3, respectively, along with the corresponding PDB-IDs.

Taken together, our CAP framework proposes a more unified approach. It uses a purely sequence-based interaction embedding, built from learned residue and nucleotide descriptors, so it requires neither three-dimensional structures, subclass labels, nor hand-crafted interface features. Furthermore, the existing models in literature run a separate regression model for every DNA/RNA subclass steps that complicate the workflow and risk information loss. Moreover, the datasets used in this study have been carefully curated to include only bona-fide DNA/RNA-binding proteins and complexes containing exactly one protein chain and one DNA/RNA chain. By removing mixed or multifunctional assemblies, the model sees a cleaner and functionally coherent signal with spurious inter-protein contacts that do not contribute to the reported affinity being eliminated. A simple ablation showed that protein-only models achieved higher correlation than DNA-only models, while the combined CAP vector performed best. This is consistent with protein interface chemistry being the primary determinant of affinity and with ESM2 embeddings encoding rich biochemical context, while the facet-based nucleotide descriptors add complementary local structure. In practice, facet-based nucleotide descriptors compute in milliseconds per complex on CPU, whereas ESM2-t36 embeddings, run as one job per protein, require seconds to about one minute depending on sequence length. Thus, CAP’s feature generation is lightweight, with protein embedding dominating the cost.

## 3. Datasets

In this section, we give a brief insight about the datasets used in this study, the data collection and curation. Dataset S186 is given by Shen *et al* [11]. It contains mostly protein-DNA complexes but has a few protein-RNA complexes as well. We created a protein-RNA dataset S142 and a protein-nucleic acid dataset S322. Both datasets were assembled by merging our own curated complexes from PDBbind-v2020 with some of the complexes used in the PRA-Pred study [37]. We followed the filtering pipeline of Shen *et al* [11], with several additional steps to improve consistency and reliability. First, all non-standard bases were removed, retaining only *A*, *C*, *G* and *T* for DNA, and *A*, *C*, *G* and *U* for RNA. Complexes having at least one nucleotide were also kept. Although, a subset of the S142 and S322 complexes also appears in other studies (e.g. PRdeltaGPred [36] and PredPRBA [35]), all retained entries passed our independent quality checks. Finally, an additional filter applied to the RNA subsets of both datasets removed systems that do not involve bona-fide RNA-binding proteins, ensuring that the remaining complexes represent genuine RBP-RNA interactions rather than incidental co-crystallizations. RNA-binding proteins often belong to well-studied families such as RNA-recognition motifs (RRMs), K-homology (KH) domains, and double-stranded RNA-binding domains (dsRBDs) [44]. These folds rely on recurring chemical strategies: clusters of lysine and arginine neutralize the phosphate backbone, while aromatic side chains stack against exposed bases or the ribose 2^′^-hydroxyl [44]. Because those interaction chemistries are reused across many RBPs, the overall contact signatures are comparatively uniform. We, therefore, restrict our RNA dataset to bona-fide RBPs and group complexes by functional class, giving CAP a chemically coherent training set. DNA-binding proteins, in contrast, encompass a broader spectrum of folds including major-groove readers, backbone clamps, and helix-wrapping architectural proteins and thus, display far more heterogeneous residue-nucleotide contact patterns [45]. The same functional filtering sharpens the RNA dataset but is less beneficial for DNA, where preserving the full diversity of recognition modes is essential. Overall, this unified and rigorously filtered dataset collection underpins the evaluation of CAP.

It is evident that both datasets display a tendency toward more negative (stronger) experimental ΔG values as sequence length increases, despite sequence counts peaking at shorter lengths. An illustration of how binding free energy varies with sequence length in S142 is represented in figure 3(b). While the strand lengths range from 4 to 93ñt, we note that the mean affinity at the short end (4ñt) is −7.05 kcal mol^−1^, whereas the longest RNAs (93ñt) bind much more strongly ($\Delta G\;=\;-12.27\;kcal\;{mol}^{-1}$). Moreover, we also observe that most RNA complexes (107) fall in the 4 to 14ñt interval with a mean $\Delta G\;=\;-9.24\;kcal\;{mol}^{-1}$. For the S322 dataset in figure 3(c), the shortest sequence in the dataset has experimental free-energy change spanning from −10.56 to −4.25 kcal mol^−1^, with a mean of −7.05 kcal mol^−1^. Conversely, the longest sequence in S322 is 93ñt and shows a single measured value of $\Delta G\;=\;-12.27\;kcal\;{mol}^{-1}$. The mean ΔG for the length interval is −9.15 kcal mol^−1^. Figure 3(d) juxtaposes the experimental and CAP-predicted binding affinities for the S186, S142, and S322 datasets. To aid visual comparison, the points from each dataset are rescaled to the same numerical range and plotted in separate, equally sized bands.

## 4. Methods

In this section, we provide an overview of PSRT. Next, we describe the vectorization of persistent commutative algebra along with natural-language processing (NLP) method and model interpretability. ML models and model evaluation metrics are also provided.

### 4.1. PSRT

PSRT introduces a new framework for studying data geometry through combinatorial commutative algebra [39]. It maps input data to a simplicial complex made up of vertices, edges, triangles, and cells of higher dimension, thereby preserving both topological and combinatorial characteristics. A filtration records how these characteristics emerge and persist across different spatial scales, yielding algebraic invariants such as persistent facet ideals and adding an algebraic viewpoint to multiscale data analysis.

Let Δ be a simplicial complex on the vertex set $V=\left\{x_{1},\ldots ,x_{n}\right\}$, and let ${\left(\Delta^{t}\right)}_{t\in R}$ be a filtration such that $\Delta^{s}\subseteq \Delta^{t}$ whenever s⩽t. Fix a field k, and consider the polynomial ring $S\;:=k\left[x_{1},\ldots ,x_{n}\right]$. To each $\Delta^{t}$, we associate the corresponding SR ideal $I^{t}\subseteq S$, defined by

(1) $$I^{t}\;:=I\left(\Delta^{t}\right):=\left\langle x_{i_{1}}\cdots x_{i_{r}}|\left\{x_{i_{1}},\ldots ,x_{i_{r}}\right\}\notin \Delta^{t}\right\rangle .$$

This construction yields a descending sequence of ideals as t increases.

Each ideal $I^{t}$ admits a canonical primary decomposition into monomial prime ideals of the form

$$I^{t}=\underset{\sigma \in ℱ\left(\Delta^{t}\right)}{⋂}P_{\sigma },\;\text{where}\;P_{\sigma }:=\left(x_{i}|x_{i}\notin \sigma \right)\subseteq S,$$

and $ℱ\left(\Delta^{t}\right)$ denotes the set of facets of $\Delta^{t}$. The ideals $P_{\sigma }$ are referred to as the facet ideals at level t.

Let

$${𝒫}^{t}\;:=\left\{P_{\sigma }|\sigma \in ℱ\left(\Delta^{t}\right)\right\}$$

denote the set of facet ideals associated to $\Delta^{t}$. To analyze the structure of ${𝒫}^{t}$ by dimension, for each i≥0, define the graded stratification

$${𝒫}_{i}^{t}:=\left\{P_{\sigma }\in {𝒫}^{t}|dim(\sigma )=i\right\},$$

so that

$${𝒫}^{t}=\overset{dim\left(\Delta^{t}\right)}{\underset{i=0}{⨆}}{𝒫}_{i}^{t}.$$

In analogy with persistent homology, we define the notion of persistent facet ideals and their associated facet persistent numbers. A facet ideal $P_{\sigma }\in {𝒫}_{i}^{t}$ is said to persist to level $t^{\prime }>t$ if $P_{\sigma }\in {𝒫}_{i}^{t^{\prime }}$. The set of such persistent i-dimensional facet ideals is denoted by

$${𝒫}_{i}^{t,t^{\prime }}:={𝒫}_{i}^{t}\cap {𝒫}_{i}^{t^{\prime }}.$$

The corresponding facet persistent number is defined as

$${ℱ}_{i}^{t,t^{\prime }}:=\left|{𝒫}_{i}^{t,t^{\prime }}\right|,$$

which counts the number of i-dimensional facet ideals common to both levels t and $t^{\prime }$.

The collection ${\left\{{ℱ}_{i}^{t,t^{\prime }}\right\}}_{i,t,t^{\prime }}$ defines a graded combinatorial invariant that captures the evolution of the minimal prime decompositions of the SR ideals across the filtration. These invariants quantify the persistence of i-dimensional facet ideals and may be regarded as an algebraic counterpart to topological barcodes in persistent homology.

In the special case where $t=t^{\prime }$, the function

$$t\mapsto {ℱ}_{i}^{t,t}$$

is referred to as the facet curve of degree i. It records the number of i-dimensional facet ideals appearing in the minimal prime decomposition of $I^{t}$ at each level t.

Furthermore, the normalized quantity

$$\frac{{ℱ}_{i}^{t,t}}{t}$$

is called the persistence rate of the i th facet persistent number at time t, and serves as a measure of the density of i-dimensional prime components within the decomposition of $I^{t}$.

### 4.2. Illustrative example

#### Square pyramid.

**Figure 4.**
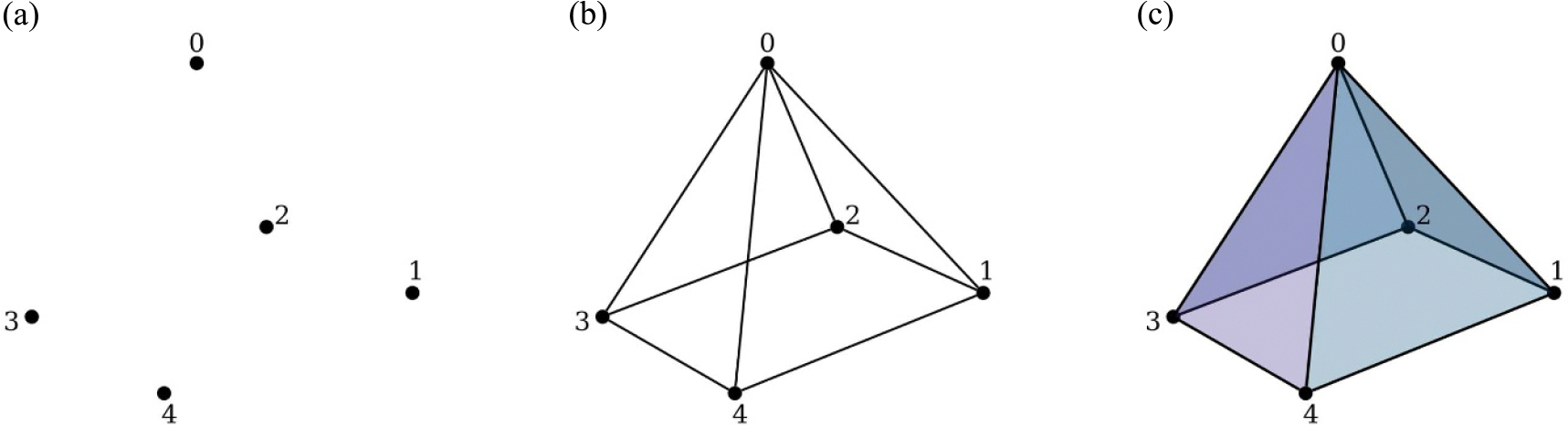
Facet filtration of a square pyramid. (a) Vertices as isolated components counted by $P_{0}$. (b) Edges as one-dimensional facets counted by $P_{1}$. (c) Two-simplices (side faces) counted by $P_{2}$, illustrating the formation of triangular faces as the filtration progresses.

Let Δ be the simplicial complex on the vertex set V={0,1,2,3,4}, illustrated in figure 4. The base is the four-cycle on {1,2,3,4} with edges

$$\left\{1,2\right\},\;\left\{2,3\right\},\;\left\{3,4\right\},\;\left\{4,1\right\},$$

and each base vertex is connected to the apex 0 via the edges

$$\left\{0,i\right\}\;for\;i\;\in \;\left\{1,2,3,4\right\}.$$

The maximal two-faces (facets) are the four triangles

$$\left\{0,1,2\right\},\;\left\{0,2,3\right\},\;\left\{0,3,4\right\},\;\left\{0,4,1\right\},$$

so combinatorially Δ is the boundary of a square pyramid.

At the vertex-only level, the five vertices form isolated components, so the facet-count vector $\left(P_{0},P_{1},P_{2}\right)$ takes the value (5,0,0); when all edges are present but no two-simplices have been added, each of the eight edges is a one-dimensional facet, giving (0,8,0); and in the full complex all four side faces appear as two-simplices and every edge is contained in some triangle, so the facet counts are (0,0,4), indicating that facet mass flows from isolated vertices ($P_{0}$) to facet edges ($P_{1}$) and then to facet two-simplices ($P_{2}$) as the filtration parameter increases. The corresponding facet-rate curves are obtained by rescaling these counts, dividing the number of active *d*-dimensional facets at filtration value t by t, which provides a multiscale summary of how facet mass moves from 0-, to 1-, to 2-dimensional simplices as the filtration progresses.

### 4.3. 1-mer algebra

In this section, we present the specialization of the *k*-mer algebra framework to the case k=1. This method derives an algebraic structure through the SR construction, based on the positional distribution of individual nucleotides. Let 𝒜 be {A,C,G,T/U}, and let

$$S=s_{1}s_{2}\cdots s_{N}\in {𝒜}^{N}$$

be a sequence of length N. For each letter a∈𝒜, define the indicator function

$$\delta_{a}:𝒜\to \{0,1\},\;\delta_{a}(b)=\left\{\begin{array}{ll} 1, & b=a,\\ 0, & b\neq a. \end{array}\right.$$

The set of positions at which a occurs in S is

$$S^{a}=\left\{i\in \{1,\ldots ,N\}|\delta_{a}\left(s_{i}\right)=1\right\}\subset N.$$

We view $S^{a}\subset R$ as a one-dimensional input data. Define the corresponding pairwise distance matrix

$$D^{a}={\left(d_{ij}^{a}\right)}_{i,j\in S^{a}}\in R^{\left|S^{a}\right|\times \left|S^{a}\right|},\;d_{ij}^{a}=|i-j|.$$

Fix a filtration interval $\left[t_{0},t_{1}\right]\subset R_{\geq 0}$. For each $t\in \left[t_{0},t_{1}\right]$, construct the Vietoris–Rips complex on $S^{a}$ at scale t and compute its algebraic invariants. Denote by

$$v_{a}^{t}={\left(v_{i}^{t}(a)\right)}_{i\in N}$$

the vector of algebraic invariants in each dimension i∈N (e.g. facet vectors ℱ, and facet rate vectors.). Restricting to a fixed scale t, we write

$$v_{a}:={\left(v_{i}(a)\right)}_{i\in N}.$$

Finally, the full 1-mer representation of S is obtained by concatenation overall a∈𝒜:

$$v_{S}^{1}\;:=\left(v_{a}|a\in 𝒜\right)={\left(v_{S,i}^{1}\right)}_{i\in N},$$

where $v_{S,i}^{1}\;:=\left(v_{i}(A),v_{i}(C),v_{i}(G),v_{i}(T/U)\right)$ is the vector of dimension-i features computed overall 1-mers in S. We refer to $v_{S}^{1}$ as the CAP-vector representation of S at k=1, which encodes the spatial distribution of each symbol via persistent algebraic invariants.

### 4.4. Vectorization of persistent commutative algebra

To capture the intrinsic structure of nucleic acid sequence, we encode every sequence as a one-dimensional input data, where each point indicates the position of a mononucleotide (i.e. *k*-mers of length 1). To ensure consistency, we applied a uniform grid of fifty equally spaced filtration thresholds, r=0,1,2,...,49, to every *k*-mer input. A Vietoris–Rips filtration is then applied using these fixed radii across all samples. This setup generates uniformly aligned facet-count curves and their corresponding rate profiles, each sampled at fifty evenly spaced filtration values. As a result, we derive two parallel families of features with a filtration design that captures structural regularities while suppressing noise and guarding against outliers.

For each sequence, we identify the positions of all four mononucleotides (A, C, G, T/U), forming four separate inputs. To each input, we apply persistent commutative algebra modeling, using persistent facet ideals to measure the algebraic complexity of interaction structures across scales. For every mononucleotide-specific cloud, we compute 50-dimensional facet curves in dimensions 0, 1, and 2, along with 50-dimensional rate curves capturing the change in facet growth across the filtration. This yields 150 features from the facet counts and 150 from the facet rates, resulting in 300 features per nucleotide. Concatenating the features across all four nucleotide types produces a 1200-dimensional feature vector that summarizes sequence-derived structural patterns. This coherent representation of simplicial structure and connectivity evolution across an identical filtration range yields a rich input for our binding-affinity model.

### 4.5. Transformer-based protein language model

Recent advances in NLP provide powerful sequence-based insights for molecular biosciences. We harness these techniques to augment our PSRT framework for predicting protein-nucleic acid binding affinities. While PSRT analyzes nucleic acid sequences, NLP captures patterns directly from amino acid sequences.

To complement the commutative-algebraic descriptors of DNA/RNA, we incorporate protein sequence information using a state-of-the-art transformer-based language model, ESM2 [15]. This model processes the raw amino acid sequence and outputs 2560-dimensional high-level embeddings by aggregating across 36 transformer layers. The final feature matrix for each protein-nucleic acid complex is constructed by concatenating the 1200-dimensional PCA-derived nucleotide features with the 2560-dimensional ESM2-based protein features, resulting in a unified 3760-dimensional representation. These composite features are used to train and evaluate CAP on our dataset. An illustration of the workflow in CAP is demonstrated in figure 1.

### 4.6. ML modeling

We train our regression models with ten-fold cross validation (CV) combined with gradient boosting decision trees (GBDT), implemented in Python via the <mono@space>scikit-learn</mono@space> library(v1.3.2). GBDT is valued for its resistance to overfitting, limited sensitivity to hyperparameter tuning, and straightforward deployment. It assembles many shallow decision trees generated from bootstrap samples of the training data and aggregates their outputs, so the ensemble corrects errors that any single tree might make. We supply the algorithm separately with PSRT-derived molecular descriptors and transformer-based descriptors; the hyperparameter settings are listed in table 3.

### 4.7. Evaluation metrics

To quantitatively evaluate the performance of our binding affinity prediction models, we employ the *PCC*, defined as:

$$PCC(x,y)=\frac{\sum_{m=1}^{M}\;\left(y_{m}^{e}-{\overset{‾}{y}}^{e}\right)\left(y_{m}^{p}-{\overset{‾}{y}}^{p}\right)}{\sqrt{\sum_{m=1}^{M}\;{\left(y_{m}^{e}-{\overset{‾}{y}}^{e}\right)}^{2}\sum_{m=1}^{M}\;{\left(y_{m}^{p}-{\overset{‾}{y}}^{p}\right)}^{2}}},$$

where $y_{m}^{e}$ and $y_{m}^{p}$ denote the experimental and predicted binding affinity values for the mth sample, respectively, and ${\overset{‾}{y}}^{e}$ and ${\overset{‾}{y}}^{p}$ are their corresponding mean values.

We also report the RMSE, which is computed as:

$$RMSE=\sqrt{\frac{1}{n}\sum_{m=1}^{M}\;{\left(y_{m}^{e}-y_{m}^{p}\right)}^{2}},$$

where $y_{m}^{e}$ and $y_{m}^{p}$ represent the experimental and predicted binding affinity values for the mth sample, respectively.

The above two metrics are employed to assess the performance of our ML models on all datasets. The original labels for these datasets are given as $pK_{d}$ values, which are converted to binding free energies (in kcal mol^−1^) by multiplying by a constant factor of 1.3633. Our models achieve reasonable RMSE values across all three datasets.

## 5. Model interpretability

Topological techniques like persistent homology (PH) [46] and persistent Laplacian theory [47] have emerged as powerful tools in ML frameworks. Although, our persistent commutative algebra technique is built on the same simplicial-complex scaffold of PH, it unravels the combinatorial activity by shifting the focus from a global view to a fine-grained, local view across the same filtration. Moreover, in comparison with persistent Laplacian theory [47], which can be computationally intensive in terms of analyzing eigenvalues of large Laplacian matrices, our algebraic construction sidesteps matrix diagonalization and scales more efficiently. Furthermore, algebraic graph methods work on the 1-skeleton (nodes and edges) using adjacency/Laplacian matrices or graph polynomials/kernels, so they mainly capture pairwise connectivity at a given scale. CAP instead models higher-order simplex structure (edges, triangles, higher faces) across scales, providing compact counts and rates that resolve local changes beyond pairwise summaries. Each of these varying paradigms offers its own unique advantages, making them suitable for different data regimes and analytical goals.

**Figure 5.**
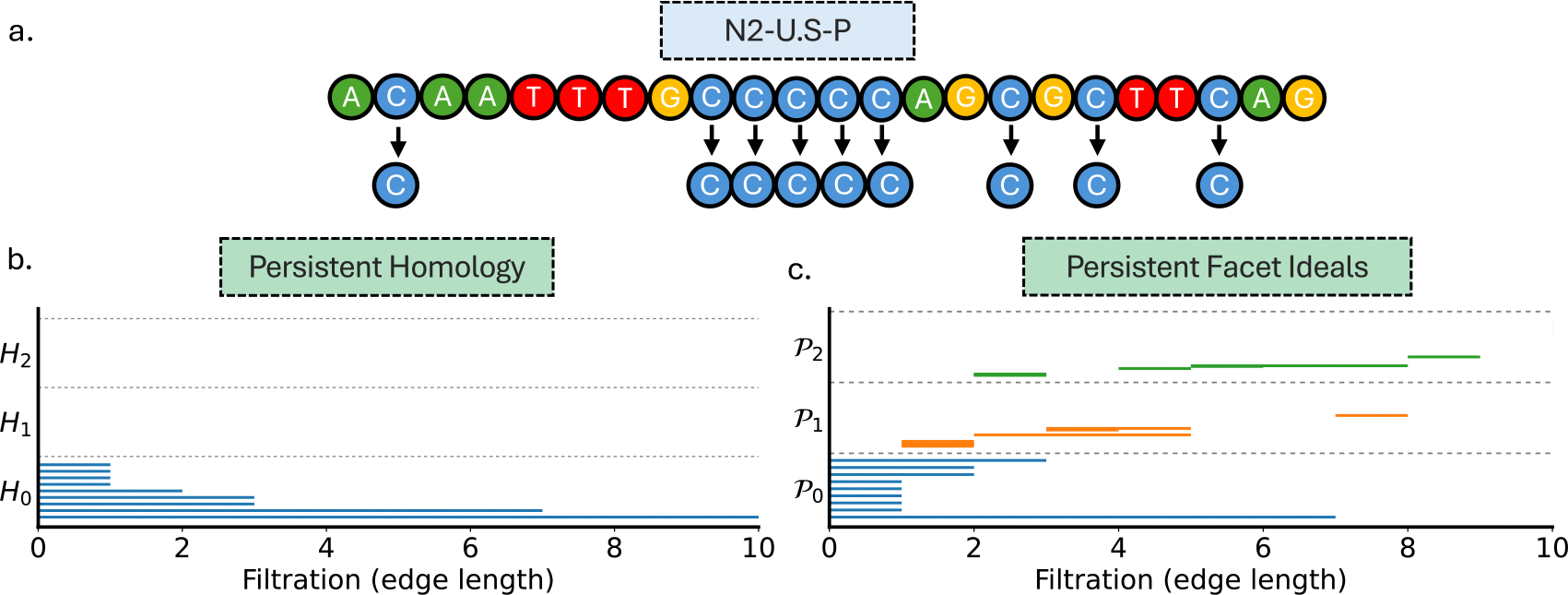
Comparison between persistent homology and persistent Stanley–Reisner invariants. (a) The N2-U.S-P primer sequence, with the positions of the nucleotide C marked. (b) The persistent homology barcodes computed from the input set formed by the positions of C, representing topological features in the spatial distribution. (c) The persistent facet barcodes derived of the same input set, encoding combinatorial face-level patterns rather than topological invariants and reflecting the algebraic invariants of maximal simplices under the same induced filtration.

In this section, we demonstrate how a simplicial complex evolves distinctively under a growing filtration parameter for PH and persistent SR approach. For this purpose, we analyze the N2-U-S-P primer sequence as shown in figure 5(a). The 23-nucleotide primer contains several cytosines that we have highlighted; only these C residues are used to build the one-dimensional input set in this example, placing each point at its sequence index (positions 2, 9, 10, 11, 12, 13, 16, 18, and 21). This resulting input set is analyzed using both persistent homology and persistent facet ideals.

Figure 5(b) shows the barcodes from the persistent homology analysis, which records the appearance and disappearance of global features—connected components, loops, and voids—as the Vietoris–Rips filtration radius r increases. For this cytosine-based one-dimensional input, the $H_{0}$ barcode comprises blue bars, each initially representing an isolated cytosine at r=0. As the filtration expands, these points progressively merge, reducing the number of separate clusters. Notably, no bars appear in the $H_{1}$ or $H_{2}$ lanes, indicating that higher-dimensional features such as loops and cavities are not formed in this case. On the contrary, figure 5(c) shows barcodes derived from the persistent facet ideals applied to the same filtration data. The zeroth-dimensional panel $P_{0}$ barcodes (blue bars) show the appearance of isolated components. The edges appear around r=1 in the first-dimensional panel $P_{1}$ (orange bars) which was entirely missed by persistent homology. Similarly, the second-dimensional panel $P_{2}$ (green bars) captures the birth and death of 2-simplices around r=2, indicating the formation of triangular faces in the structure as the filtration progresses.

Persistent homology and persistent facet ideals both analyze how a simplicial complex evolves under a growing filtration parameter, but they focus on different features. Unlike persistent homology which is summarized by the Betti-number barcodes, persistent facet ideals directly capture local simplex activities (vertices, edges, triangles, and higher-order structures) throughout the filtration process. The filtration process involves gradually increasing a radius parameter r to monitor the formation and dissolution of topological features encoded via facet ideals. For example, the $H_{0}$ barcode records the number of connected components: it begins at the total number of vertices and decreases by one each time an edge appears, reaching a single component when the complex becomes fully connected. In contrast, the facet-ideal count $P_{0}$ measures the number of isolated vertices (0-simplices not yet incident to any edge) and thus falls to zero as soon as every vertex participates in at least one edge. For example, with four vertices, initially $H_{0}\;=\;4$ and $P_{0}\;=\;4$; after adding one edge between two vertices, the complex has three components so $H_{0}\;=\;3$, while only two vertices remain isolated so $P_{0}\;=\;2$; if additional edges are added so that every vertex has at least one incident edge but the graph still has two components, then $H_{0}\;=\;2$ while $P_{0}\;=\;0$. This illustrates the key distinction: $\beta_{0}$ (via $H_{0}$) tracks how many pieces the space has, whereas $P_{0}$ tracks how many vertices are still completely unused, so the two barcodes capture complementary aspects of the same filtration. More broadly, persistent homology emphasizes the emergence of homology classes that change the global topology, while persistent facet ideals specialize in capturing precise local combinatorial events, which can improve interpretability and downstream machine-learning performance on biomolecular data.

## 6. Conclusion

Commutative algebra, traditionally central to fields like algebraic geometry and number theory, has remained largely untapped in data-driven and biological research. Recent work by Suwayyid and Wei [39] changes this by integrating SR theory with multiscale analysis, opening a new avenue for nonlinear algebraic techniques in data science. Initial studies, such as those on protein-ligand binding affinity [40], attest to the power of the PSRT framework for enhancing ML models.

In this work, we present CAP, a framework for protein-nucleic acid binding affinity prediction that integrates PSRT-based *k*-mer algebra descriptors of DNA sequences and transformer-derived ESM2 embeddings of protein sequences. We evaluate the performance of our model on the S186 dataset with an additional test on newly curated datasets S142 and S322. CAP is trained with a single gradient-boosting regression tree model to provide variance across splits without the need for iterative feature pruning or subclass-specific retraining. Our findings deliver strong and generalizable predictions with *PCC* of 0.705 with RMSE of 1.79 kcal mol^−1^ outperforming SVSBI [11] (0.669/1.98 kcal mol^−1^). Furthermore, CAP attains a *PCC* of 0.653 and an RMSE of 2.18 kcal mol^−1^ on S142, while its performance improves on S322, reaching a *PCC* of 0.669 with an RMSE of 2.0 kcal mol^−1^. These results demonstrate the robustness of CAP in jointly capturing informative DNA k-mer algebra signatures and protein ESM2 embeddings, thereby enabling reliable prediction of protein-nucleic acid binding affinities. Finally, because CAP’s inputs are limited to primary sequences, the framework is inherently scalable: it can be applied to any protein-DNA/RNA pair with minimal preprocessing and is readily extensible to genome-scale studies. In addition to this, the proposed commutative algebraic methodology can not only be applied to the study of other biological sequences, such as phylogenetic analysis and protein sequence representation but it can also be generalized to the analysis of other sequential data in science and engineering.

## Supplementary Material

SI

Supplementary material for this article is available online

## Figures and Tables

**Table 1.** Comparison of prediction performance between existing SVSBI model and our model, CAP, on S186 for protein-nucleic acid binding affinity prediction. Reported metrics include Pearson correlation coefficient (*PCC*) and RMSE values in kcal mol^−1^. All results are averaged over twenty independent runs with different random seeds, and the average metric values are reported. Bold values indicate the performance of the proposed CAP model.

Model	PCC	RMSE (kcal mol^−1^)

SVSBI [11]	0.669	1.98^a^
**CAP**	**0.705**	**1.79**

a The original RMSE of 1.45 reported by Shen *et al* [11] was not converted into kcal mol^−1^; here we apply the factor 1.3633 to obtain 1.98 kcal mol^−1^.

**Table 2.** Prediction performance of CAP on S142 and S322 datasets (mean ± standard deviation over 10-fold CV).

Dataset	PCC	RMSE (kcal mol^−1^)

S142	0.665 ± 0.02	2.16 ± 0.05
S322	0.669 ± 0.01	2.00 ± 0.02

**Table 3.** Hyperparameters used in the gradient boosting regression model within a scikit-learn pipeline. StandardScaler is applied prior to model training.

No. of estimators	Max depth	Min. samples split	Learning rate
10 000	7	3	0.01

Max features	Subsample size	Random state	Standardization
Square root	0.7	Fixed (seeded)	Yes (StandardScaler)

## Data Availability

The data that support the findings of this study are openly available at the following URL/DOI: https://github.com/mzia-s/CAP.

## References

[1] Bertoldo JB, Müller S and Hüttelmaier S 2023 RNA-binding proteins in cancer drug discovery Drug Discovery Today 28 103580 37031812 10.1016/j.drudis.2023.103580

[2] Kisby GE, Wilson DM and Spencer PS 2024 Introducing the role of genotoxicity in neurodegenerative diseases and neuropsychiatric disorders Int. J. Mol. Sci. 25 7221 39000326 10.3390/ijms25137221 PMC11241460

[3] Stockley PG 2009 Filter-binding assays DNA-Protein Interactions: Principles and Protocols 3rd edn pp 1–14

[4] Zhang C, Liu S, Zhu Q and Zhou Y 2005 A knowledge-based energy function for protein-ligand, protein- protein and protein-DNA complexes J. Med. Chem. 48 2325–35 15801826 10.1021/jm049314d

[5] Nithin C, Mukherjee S and Bahadur RP 2019 A structure-based model for the prediction of protein–RNA binding affinity RNA 25 1628–45 31395671 10.1261/rna.071779.119 PMC6859855

[6] Yin S, Biedermannova L, Vondrasek J and Dokholyan NV 2008 Medusascore: an accurate force field-based scoring function for virtual drug screening J. Chem. Inf. Model. 48 1656–62 18672869 10.1021/ci8001167 PMC2665000

[7] Bitencourt-Ferreira G and Filgueira de Azevedo W Jr 2019 Machine learning to predict binding affinity Docking Screens for Drug Discovery (Springer) pp 251–73 10.1007/978-1-4939-9752-7_16 31452110

[8] Hafner M 2010 Transcriptome-wide identification of RNA-binding protein and microrna target sites by PAR-CLIP Cell 141 129–41 20371350 10.1016/j.cell.2010.03.009 PMC2861495

[9] Zhao H, Yang Y and Zhou Y 2011 Structure-based prediction of RNA-binding domains and RNA-binding sites and application to structural genomics targets Nucleic Acids Res. 39 3017–25 21183467 10.1093/nar/gkq1266 PMC3082898

[10] Liu Z-P and Miao H 2016 Prediction of protein-RNA interactions using sequence and structure descriptors Neurocomputing 206 28–34

[11] Li Shen, Feng H, Qiu Y and Wei G-W 2023 SVSBI: sequence-based virtual screening of biomolecular interactions Commun. Biol. 6 536 37202415 10.1038/s42003-023-04866-3 PMC10195826

[12] Tuszynska I and Bujnicki JM 2011 Dars-RNP and quasi-RNP: new statistical potentials for protein-RNA docking BMC Bioinform. 12 1–16 10.1186/1471-2105-12-348 PMC3179970 21851628

[13] Setny P and Zacharias M 2011 A coarse-grained force field for protein–RNA docking Nucleic Acids Res. 39 9118–29 21846771 10.1093/nar/gkr636 PMC3241652

[14] Iwakiri J, Hamada M, Asai K and Kameda T 2016 Improved accuracy in RNA–protein rigid body docking by incorporating force field for molecular dynamics simulation into the scoring function J. Chem. Theory Comput. 12 4688–97 27494732 10.1021/acs.jctc.6b00254

[15] Lin Z 2023 Evolutionary-scale prediction of atomic-level protein structure with a language model Science 379 1123–30 36927031 10.1126/science.ade2574

[16] Song Y and Wang L 2024 Multiobjective tree-based reinforcement learning for estimating tolerant dynamic treatment regimes Biometrics 80 ujad017 38364801 10.1093/biomtc/ujad017 PMC10871869

[17] Lo Y-C, Rensi SE, Torng W and Altman RB 2018 Machine learning in chemoinformatics and drug discovery Drug Discovery Today 23 1538–46 29750902 10.1016/j.drudis.2018.05.010 PMC6078794

[18] Cang Z and Wei G-W 2017 TopologyNet: topology based deep convolutional and multi-task neural networks for biomolecular property predictions PLoS Comput. Biol. 13 e1005690 28749969 10.1371/journal.pcbi.1005690 PMC5549771

[19] Papamarkou T 2024 Position: topological deep learning is the new frontier for relational learning Proc. Mach. Learn. Res.235 39529 40196046 PMC11973457

[20] Nguyen DD, Cang Z and Wei G-W 2020 A review of mathematical representations of biomolecular data Phys. Chem. Chem. Phys. 22 4343–67 32067019 10.1039/c9cp06554g PMC7081943

[21] Rana K 2023 The Geometry and Topology of Landslides (Rochester Institute of Technology)

[22] Wang Y, Liu X, Zhang Y, Wang X and Xia K 2025 Join persistent homology (JPH)-based machine learning for metalloprotein–ligand binding affinity prediction J. Chem. Inform. Model. 65 2785–93 10.1021/acs.jcim.4c02309 40082211

[23] Nguyen DD, Cang Z, Wu K, Wang M, Cao Y and Wei G-W 2019 Mathematical deep learning for pose and binding affinity prediction and ranking in D3R grand challenges J. Comput.-Aided Mol. Des. 33 71–82 30116918 10.1007/s10822-018-0146-6 PMC7163798

[24] Nguyen DD, Gao K, Wang M and Wei G-W 2020 MathDL: mathematical deep learning for D3R grand challenge 4 J. Comput. Aided Mol. Des. 34 131–47 31734815 10.1007/s10822-019-00237-5 PMC7376411

[25] Hozumi Y and Wei G-W 2024 Revealing the shape of genome space via K-mer topology (arXiv:2412.20202)

[26] Liu J, Shen Li, Chen D and Wei G-W 2025 Topological sequence analysis of genomes: delta complex approaches (arXiv:2507. 05452)

[27] Liu J, Shen Li, Zia M and Wei G-W 2025 Topological sequence analysis of genomes: category approaches (arXiv:2507.08043)

[28] Zhao H, Yang Y and Zhou Y 2010 Structure-based prediction of DNA-binding proteins by structural alignment and a volume-fraction corrected DFIRE-based energy function Bioinformatics 26 1857–63 20525822 10.1093/bioinformatics/btq295 PMC2905551

[29] Rastogi C 2018 Accurate and sensitive quantification of protein-DNA binding affinity Proc. Natl Acad. Sci.115 E3692–701 10.1073/pnas.1714376115 29610332 PMC5910815

[30] Barissi S, Sala A, Wieczór M, Battistini F and Orozco M 2022 Dnaffinity: a machine-learning approach to predict DNA binding affinities of transcription factors Nucleic Acids Res. 50 9105–14 36018808 10.1093/nar/gkac708 PMC9458447

[31] Yang W and Deng L 2020 PreDBA: a heterogeneous ensemble approach for predicting protein-DNA binding affinity Sci. Rep. 10 1278 31992738 10.1038/s41598-020-57778-1 PMC6987227

[32] Harini K, Kihara D and Michael Gromiha M 2023 PDA-PRED: predicting the binding affinity of protein-DNA complexes using machine learning techniques and structural features Methods 213 10–17 36924867 10.1016/j.ymeth.2023.03.002 PMC10563387

[33] Yang S, Gong W, Zhou T, Sun X, Chen L, Zhou W and Li C 2023 empDBA: protein-DNA binding affinity prediction by combining features from binding partners and interface learned with ensemble regression model Brief. Bioinform. 24 bbad192 37193676 10.1093/bib/bbad192

[34] Yang X, Li H, Huang Y and Liu S 2013 The dataset for protein–RNA binding affinity Protein Sci. 22 1808–11 24127340 10.1002/pro.2383 PMC3843634

[35] Deng L, Yang W and Liu H 2019 PredPRBA: prediction of protein-RNA binding affinity using gradient boosted regression trees Front. Genet. 10 637 31428122 10.3389/fgene.2019.00637 PMC6688581

[36] Hong X, Tong X, Xie J, Liu P, Liu X, Song Q, Liu S and Liu S 2023 An updated dataset and a structure-based prediction model for protein–RNA binding affinity Proteins: Struct. Funct. Bioinform. 91 1245–53 10.1002/prot.26503 37186412

[37] Harini K, Sekijima M and Michael Gromiha M 2024 PRA-PRED: structure-based prediction of protein-RNA binding affinity Int. J. Biol. Macromol. 259 129490 38224813 10.1016/j.ijbiomac.2024.129490

[38] Eisenbud D 2013 Commutative Algebra: With a View Toward Algebraic Geometry vol 150 (Springer)

[39] Suwayyid F and Wei G-W 2025 Persistent Stanley–Reisner theory (arXiv:2503.23482) 10.3934/fods.2025009 PMC13441236 42559569

[40] Feng H, Suwayyid F, Zia M, Wee J, Hozumi Y, Chen C-L and Wei G-W 2025 CAML: Commutative algebra machine learning—a case study on protein–ligand binding affinity prediction J. Chem. Inform. Model. 65 6732–43 10.1021/acs.jcim.5c00940 PMC12264937 40518771

[41] Stanley RP 2007 Combinatorics and Commutative Algebra vol 41 (Springer)

[42] Francisco CA, Mermin J and Schweig J 2014 A Survey of Stanley–Reisner Theory Connections Between Algebra, Combinatorics and Geometry (Springer) pp 209–34

[43] Peng Y and Alexov E 2017 Computational investigation of proton transfer, pKa shifts and pH-optimum of protein-DNA and protein-RNA complexes Biophys. J. 112 518a 10.1002/prot.25221 PMC9843452 27936518

[44] Corley M, Burns MC and Yeo GW 2020 How RNA-binding proteins interact with RNA: molecules and mechanisms Mol. Cell 78 9–29 32243832 10.1016/j.molcel.2020.03.011 PMC7202378

[45] Yesudhas D, Batool M, Anwar MA, Panneerselvam S and Choi S 2017 Proteins recognizing DNA: structural uniqueness and versatility of DNA-binding domains in stem cell transcription factors Genes 8 192 28763006 10.3390/genes8080192 PMC5575656

[46] Zomorodian A and Carlsson G 2004 Computing persistent homology Proc. 20th Annual Symp.on Computational Geometry pp 347–56

[47] Wang R, Nguyen DD and Wei G-W 2020 Persistent spectral graph Int. J. Numer. Methods Biomed. Eng. 36 e3376 10.1002/cnm.3376 PMC7719081 32515170

