# Supplementary material for: CAP: Commutative algebra prediction of protein-nucleic acid binding affinities
Source: Mach Learn Sci Technol. Author manuscript; Available in PMC 2026 Mar 20. (PMC13001652; doi:10.1088/2632-2153/ae29bc)
Supplement: SI [file NIHMS2148861-supplement-SI.pdf]

Supporting Information

CAP: Commutative Algebra Prediction of Protein-Nucleic Acid Binding Affinities

Mushal Zia[1], Faisal Suwayyid [1,2], Yuta Hozumi* [1]
JunJie Wee[1], Hongsong Feng[3], and Guo-Wei Wei†[1,4,5]
[1]Department of Mathematics,
Michigan State University, MI 48824, USA.
[2]Department of Mathematics,
King Fahd University of Petroleum and Minerals, Dhahran 31261, KSA.
[3]Department of Mathematics and Statistics,
University of North Carolina at Charlotte, Charlotte, NC 28223, USA
[4]Department of Electrical and Computer Engineering,
Michigan State University, MI 48824, USA.
[5]Department of Biochemistry and Molecular Biology,
Michigan State University, MI 48824, USA.

*Current address: School of Mathematics, Georgia Institute of Technology, Atlanta, GA, USA.
†Corresponding author: Guo-Wei Wei (weig@msu.edu).

Contents

1 Results: Experimental vs. Predicted Binding Affinities 2

1.1 The S186 Dataset 2

1.2 The S142 Dataset 4

1.3 The S322 Dataset 6

1 Results: Experimental vs. Predicted Binding Affinities

1.1 The S186 Dataset

Table S1: Experimental and predicted binding free energies (ΔG) for the S186 dataset [1].

PDBID	Exp BA	Pred BA	PDBID	Exp BA	Pred BA	PDBID	Exp BA	Pred BA
1HVO	-6.760	-7.227	1WET	-7.940	-11.516	2PUF	-7.769	-11.158
1QPZ	-11.704	-10.246	1QP0	-10.165	-10.955	1QP7	-8.327	-8.392
1FJX	-12.070	-12.671	1J75	-10.165	-9.392	1JFS	-11.803	-10.957
1J5K	-7.529	-9.805	1PO6	-9.276	-9.696	1P51	-11.528	-11.390
1QZG	-8.614	-9.464	1OSB	-9.754	-9.754	1HVN	-7.227	-6.758
1BDH	-8.242	-8.118	1QP4	-10.906	-11.221	1QQB	-7.769	-8.664
1TW8	-12.192	-12.234	1QQA	-8.722	-8.250	1DH3	-12.070	-10.448
1JJ4	-11.991	-10.913	1FYM	-10.086	-9.559	1JH9	-11.859	-7.913
1JT0	-9.965	-10.387	1P78	-11.528	-11.555	1P71	-11.528	-11.296
1OMH	-9.754	-9.754	1S40	-12.983	-10.193	1U1L	-9.503	-8.938
1U1R	-9.053	-9.165	1U1N	-8.862	-9.677	1U1O	-9.855	-9.646
2BJC	-14.996	-10.129	1ZZI	-7.713	-9.770	2B0D	-8.242	-9.134
2GII	-12.783	-12.781	2GIE	-12.592	-12.582	2GIH	-12.252	-12.254
2ADY	-10.707	-10.494	2AC0	-10.364	-10.013	2AYB	-11.859	-11.797
2ERE	-10.256	-10.295	2GE5	-8.751	-9.147	2O6M	-10.751	-10.058
3D6Z	-8.204	-6.798	2VYE	-9.991	-9.671	3IGM	-8.590	-9.391
3HXQ	-12.572	-12.566	3EQT	-9.514	-9.864	3H15	-7.455	-8.684
2KAE	-11.039	-9.858	3MQ6	-12.572	-10.865	3JSP	-11.995	-12.031
3AAF	-8.994	-9.236	3N1L	-11.407	-11.239	3N1K	-11.236	-11.406
3R8F	-9.487	-9.775	2RRA	-7.940	-9.453	3QMI	-6.709	-6.639
2KXN	-7.700	-9.556	1U1P	-9.107	-9.274	1U1M	-8.901	-9.165
1U1K	-8.856	-9.141	1U1Q	-9.706	-9.586	2AOR	-8.242	-8.299
2AOQ	-7.438	-8.094	2I9K	-12.983	-11.832	2GIJ	-12.783	-12.781
2GIG	-12.252	-12.254	2CCZ	-9.543	-10.346	2AHI	-10.496	-10.705
2ERG	-10.364	-10.034	2AYG	-11.859	-11.765	2ES2	-8.843	-9.358
2ATA	-9.646	-10.400	2NP2	-9.897	-10.567	3D6Y	-6.799	-8.206
2VY1	-9.573	-9.888	3HXO	-12.572	-12.572	3GIB	-12.070	-9.482
3D2W	-9.355	-9.032	2KKF	-5.939	-7.859	3M9E	-9.388	-9.799
3JSO	-12.402	-11.641	3K3R	-11.966	-12.244	3N1I	-11.554	-10.859
3N1J	-11.386	-10.859	3KJP	-11.180	-9.631	3Q0B	-8.134	-9.231
3PIH	-11.890	-10.408	3QMH	-6.502	-6.823	3QMB	-7.438	-6.574

PDBID	Exp BA	Pred BA	PDBID	Exp BA	Pred BA	PDBID	Exp BA	Pred BA
3U7F	-8.482	-9.231	3RN2	-9.476	-9.011	4ATK	-11.956	-11.850
2LTT	-10.628	-10.156	4HIO	-10.467	-10.106	4HIK	-10.388	-10.374
4A76	-9.855	-9.274	4HJ7	-11.209	-10.445	4HID	-8.273	-10.424
4LJ0	-8.590	-9.187	4HT8	-9.471	-11.451	4GCK	-9.256	-9.742
4F2J	-10.364	-10.110	4GCT	-9.915	-9.872	4NI7	-13.223	-9.962
4CH1	-9.003	-9.149	4QJU	-8.768	-10.589	4ZBN	-13.194	-10.142
4R55	-7.876	-9.658	4S0N	-10.751	-9.311	4ZSF	-11.639	-10.816
4RKG	-6.258	-9.099	5A72	-9.916	-10.327	5DWA	-12.332	-10.915
5K83	-7.913	-6.797	5ITH	-10.256	-9.518	5W9S	-9.543	-8.161
5YI3	-8.534	-9.730	5XFP	-8.072	-9.584	5MEY	-9.265	-9.084
5MEZ	-9.001	-9.356	5W2M	-7.084	-7.453	5K17	-6.891	-9.412
6MG1	-10.276	-11.106	5VMV	-12.769	-10.045	6CNQ	-8.242	-9.063
6FQP	-9.229	-9.342	5WWF	-9.029	-9.040	6FQQ	-8.739	-9.349
5ZVB	-5.990	-5.940	5ZVA	-5.809	-6.266	5MPF	-9.605	-9.887
6G1L	-12.332	-11.459	6IIQ	-8.024	-9.616	6IIR	-7.570	-9.127
3ON0	-11.341	-10.466	4HQU	-14.586	-11.900	4HQX	-12.162	-13.442
4A75	-9.232	-9.818	3QSU	-10.511	-10.019	4HJ8	-10.440	-8.511
4HIM	-10.132	-10.470	4HJ5	-9.817	-10.383	4HP1	-7.028	-8.751
4J1J	-8.584	-10.111	4NM6	-8.140	-9.119	4GCL	-9.278	-9.826
3ZPL	-11.751	-10.355	3ZH2	-10.057	-10.866	4HT4	-11.039	-9.790
4LNQ	-8.011	-9.152	4LJR	-10.244	-10.216	4R56	-9.370	-6.700
4TMU	-12.030	-7.978	4R22	-10.819	-10.223	4Z3C	-7.661	-8.187
3WPC	-10.496	-11.437	3WPD	-11.619	-10.389	2N8A	-9.573	-9.650
5HRT	-12.114	-10.186	5T1J	-10.526	-9.908	5VC9	-9.265	-7.710
5HLG	-8.873	-10.191	5W9Q	-8.482	-7.733	6ASB	-7.940	-9.008
6ASD	-7.661	-8.559	5K07	-6.122	-9.359	5YI2	-9.738	-8.594
6MG3	-11.449	-10.330	6FWR	-8.873	-10.244	6CNP	-8.391	-8.553
5ZD4	-10.798	-9.562	5ZMO	-9.163	-9.907	6BWY	-6.251	-9.430
6CRM	-7.264	-11.533	6CC8	-7.181	-9.183	6BUX	-5.807	-8.405
6A2I	-8.701	-7.031	5ZKI	-8.015	-10.158	6KBS	-7.713	-9.911
5ZKL	-10.479	-10.177	5ZMD	-7.407	-9.448	6ON0	-9.605	-9.688

1.2 The S142 Dataset

Table S2: Experimental and predicted binding free energies (ΔG) for the S142 dataset.

PDBID	Exp BA	Pred BA	PDBID	Exp BA	Pred BA	PDBID	Exp BA	Pred BA
2A9X	-9.303	-10.928	3BX3	-9.874	-10.332	3IRW	-14.996	-11.417
3IWN	-12.270	-13.614	3K0J	-10.992	-13.009	3QGB	-11.528	-11.324
2XS2	-10.113	-7.450	3QGC	-10.982	-10.932	4GHA	-8.590	-9.752
3V6Y	-11.464	-11.571	2LUP	-6.090	-10.548	4M59	-9.531	-12.045
2MTV	-9.526	-7.434	4RCM	-6.350	-8.934	5EIM	-8.123	-8.661
5GXH	-7.661	-9.294	5KLA	-10.334	-11.841	5EN1	-8.942	-8.889
5F5H	-9.668	-9.571	5WZK	-9.265	-10.337	5HO4	-9.462	-9.306
5U9B	-9.388	-8.770	5WZJ	-10.751	-10.317	5TF6	-11.639	-10.081
5SZE	-10.459	-8.724	5WZG	-10.218	-10.508	5M8I	-5.736	-8.548
5UDZ	-9.592	-9.059	5YTV	-8.043	-7.852	6FQ3	-8.015	-9.048
5YTX	-8.007	-8.022	5YKI	-12.030	-9.858	6DCL	-10.647	-9.134
6GD3	-5.191	-6.956	6G2K	-6.427	-6.652	6CMN	-11.727	-9.367
5YTS	-7.577	-7.648	5WWG	-9.077	-8.823	5WWF	-9.029	-8.892
5YTT	-7.394	-7.691	6FQR	-6.073	-7.340	6GX6	-7.264	-6.194
5WWE	-8.550	-9.024	6GC5	-8.435	-6.979	6RT6	-6.755	-7.770
6NOF	-10.617	-10.477	6R7B	-10.558	-9.084	6NOC	-10.490	-10.527
6NOH	-10.421	-10.602	6A6J	-9.068	-8.372	6NOD	-10.280	-11.359
6G99	-6.176	-9.008	6RT7	-7.405	-7.234	6U9X	-10.086	-9.895
6NY5	-9.952	-10.127	6GBM	-5.556	-9.515	1EC6	-7.990	-8.866
1M8Y	-9.270	-12.410	1RPU	-13.300	-9.820	1UTD	-16.890	-8.985
2B6G	-10.600	-9.754	2ERR	-12.200	-8.668	2F8K	-10.300	-9.725
2G4B	-7.830	-7.809	2KFY	-8.840	-8.056	2KG0	-7.400	-8.786
2KG1	-7.450	-8.247	2KX5	-11.400	-10.733	2KXN	-8.170	-8.775
2L41	-4.250	-7.057	2LA5	-11.500	-10.957	2LEB	-9.320	-9.455
2LEC	-9.440	-9.339	2M8D	-8.730	-8.857	2MJH	-9.670	-9.264
2MXY	-7.900	-6.836	2MZ1	-6.870	-7.886	2N82	-10.300	-9.827
2RRA	-7.940	-8.220	2RU3	-8.520	-9.256	2XC7	-7.040	-8.121
2XFM	-8.240	-7.664	2XNR	-5.400	-7.537	2ZKO	-8.050	-10.836
3BSB	-10.200	-10.686	3BSX	-11.500	-11.098	3BX2	-9.970	-9.783
3EQT	-9.520	-9.285	3GIB	-10.690	-9.402	3K49	-12.050	-10.730
3K4E	-10.800	-11.844	3K5Q	-9.300	-9.172	3K5Y	-10.540	-9.425
3K5Z	-9.380	-9.951	3K61	-8.890	-9.677	3K62	-8.740	-9.250
3K64	-9.220	-8.941	3L25	-8.175	-9.291	3LQX	-12.220	-10.107
3MDG	-7.850	-8.737	3MOJ	-13.000	-10.506	3NCU	-10.300	-9.229

PDBID	Exp BA	Pred BA	PDBID	Exp BA	Pred BA	PDBID	Exp BA	Pred BA
3NMR	-8.440	-7.356	3NNH	-6.190	-8.264	3O3I	-6.520	-7.069
3O6E	-7.070	-6.522	3Q0L	-12.300	-11.270	3Q0M	-12.500	-12.366
3Q0N	-10.500	-10.977	3Q0P	-12.800	-10.299	3Q0Q	-13.300	-12.526
3Q0R	-13.800	-12.844	3Q0S	-10.800	-12.416	3QG9	-10.600	-10.987
3U4M	-15.900	-10.326	3V71	-11.100	-10.255	3V74	-11.600	-11.382
3WBM	-9.980	-10.926	4CIO	-9.760	-8.359	4ED5	-9.080	-8.344
4ERD	-9.540	-9.409	4HT8	-9.380	-10.205	4JVH	-9.590	-9.179
4KJI	-8.940	-10.087	4LG2	-9.540	-9.117	4NL3	-7.380	-10.254
4O26	-8.200	-11.597	4OE1	-12.110	-9.503	4QI2	-9.440	-9.509
4QVC	-11.560	-10.060	4QVD	-10.120	-10.314	4R3I	-7.770	-6.755
4RCJ	-8.180	-8.213	4TUX	-10.400	-9.475	4U8T	-9.130	-7.535
4Z31	-8.790	-9.061	5DNO	-7.830	-8.431	5ELR	-6.250	-8.894
5V7C	-6.990	-8.994	5W1I	-12.790	-10.833	5WZH	-10.460	-10.535
6D12	-9.300	-10.184						

1.3 The S322 Dataset

Table S3: Experimental and predicted binding free energies (ΔG) for the S322 dataset.

PDBID	Exp BA	Pred BA	PDBID	Exp BA	Pred BA	PDBID	Exp BA	Pred BA
1HVO	-6.760	-7.223	1WET	-7.940	-11.499	2PUF	-7.769	-10.978
1QPZ	-11.704	-10.395	1QP0	-10.165	-10.829	1QP7	-8.327	-8.595
1FJX	-12.070	-12.656	1J75	-10.165	-9.183	1JFS	-11.803	-9.749
1J5K	-7.529	-7.948	1PO6	-9.276	-9.960	1P51	-11.528	-11.533
1QZG	-8.614	-9.556	1OSB	-9.754	-10.591	1HVN	-7.227	-6.756
1BDH	-8.242	-8.219	1QP4	-10.906	-11.018	1QQB	-7.769	-8.286
1TW8	-12.192	-12.227	1QQA	-8.722	-8.317	1DH3	-12.070	-10.206
1JJ4	-11.991	-10.613	1FYM	-10.086	-9.461	1JH9	-11.859	-7.513
1JT0	-9.965	-10.345	1P78	-11.528	-11.583	1P71	-11.528	-11.213
1OMH	-9.754	-10.591	1S40	-12.983	-9.850	1U1L	-9.503	-8.936
1U1R	-9.053	-9.062	1U1N	-8.862	-9.658	1U1O	-9.855	-9.707
2BJC	-14.996	-9.751	1ZZI	-7.713	-8.013	2B0D	-8.242	-9.262
2GII	-12.783	-12.776	2GIE	-12.592	-12.521	2GIH	-12.252	-12.257
2ADY	-10.707	-10.172	2AC0	-10.364	-10.105	2AYB	-11.859	-11.788
2ERE	-10.256	-10.373	2GE5	-8.751	-8.892	2O6M	-10.751	-9.809
3D6Z	-8.204	-6.798	2VYE	-9.991	-9.824	3IGM	-8.590	-8.917
3HXQ	-12.572	-12.574	3EQT	-9.514	-9.441	3H15	-7.455	-8.787
2KAE	-11.039	-10.011	3MQ6	-12.572	-10.746	3JSP	-11.995	-11.719
3AAF	-8.994	-9.069	3N1L	-11.407	-11.236	3N1K	-11.236	-11.405
3R8F	-9.487	-10.296	2RRA	-7.940	-8.951	3QMI	-6.709	-6.751
2KXN	-7.700	-8.384	1U1P	-9.107	-9.258	1U1M	-8.901	-9.141
1U1K	-8.856	-9.163	1U1Q	-9.706	-9.535	2AOR	-8.242	-8.350
2AOQ	-7.438	-8.306	2I9K	-12.983	-11.709	2GIJ	-12.783	-12.774
2GIG	-12.252	-12.259	2CCZ	-9.543	-9.584	2AHI	-10.496	-10.172
2ERG	-10.364	-10.075	2AYG	-11.859	-11.878	2ES2	-8.843	-8.857
2ATA	-9.646	-10.318	2NP2	-9.897	-10.358	3D6Y	-6.799	-8.204
2VY1	-9.573	-9.601	3HXO	-12.572	-12.576	3GIB	-12.070	-9.609
3D2W	-9.355	-8.989	2KKF	-5.939	-8.127	3M9E	-9.388	-9.825
3JSO	-12.402	-11.298	3K3R	-11.966	-12.190	3N1I	-11.554	-11.383
3N1J	-11.386	-11.553	3KJP	-11.180	-9.073	3Q0B	-8.134	-9.216
3PIH	-11.890	-10.759	3QMH	-6.502	-6.837	3QMB	-7.438	-6.471
3U7F	-8.482	-8.392	3RN2	-9.476	-8.669	4ATK	-11.956	-11.746
2LTT	-10.628	-10.365	4HIO	-10.467	-10.124	4HIK	-10.388	-10.374
4A76	-9.855	-9.249	4HJ7	-11.209	-10.307	4HID	-8.273	-10.406

PDBID	Exp BA	Pred BA	PDBID	Exp BA	Pred BA	PDBID	Exp BA	Pred BA
4LJ0	-8.590	-9.679	4HT8	-9.471	-10.618	4GCK	-9.256	-9.238
4F2J	-10.364	-10.413	4GCT	-9.915	-9.734	4NI7	-13.223	-10.421
4CH1	-9.003	-9.759	4QJU	-8.768	-10.730	4ZBN	-13.194	-10.140
4R55	-7.876	-9.748	4S0N	-10.751	-9.187	4ZSF	-11.639	-10.971
4RKG	-6.258	-8.765	5A72	-9.916	-10.035	5DWA	-12.332	-10.702
5K83	-7.913	-7.139	5ITH	-10.256	-8.939	5W9S	-9.543	-7.754
5YI3	-8.534	-9.757	5XFP	-8.072	-8.756	5MEY	-9.265	-9.248
5MEZ	-9.001	-9.730	5W2M	-7.084	-7.220	5K17	-6.891	-9.173
6MG1	-10.276	-11.093	5VMV	-12.769	-9.678	6CNQ	-8.242	-8.450
6FQP	-9.229	-9.255	5WWF	-9.029	-9.019	6FQQ	-8.739	-9.723
5ZVB	-5.990	-5.836	5ZVA	-5.809	-6.133	5MPF	-9.605	-9.268
6G1L	-12.332	-11.436	6IIQ	-8.024	-8.421	6IIR	-7.570	-8.161
3ON0	-11.341	-10.307	4HQU	-14.586	-11.704	4HQX	-12.162	-13.473
4A75	-9.232	-9.772	3QSU	-10.511	-9.455	4HJ8	-10.440	-8.520
4HIM	-10.132	-10.460	4HJ5	-9.817	-10.383	4HP1	-7.028	-8.579
4J1J	-8.584	-10.562	4NM6	-8.140	-8.975	4GCL	-9.278	-9.652
3ZPL	-11.751	-10.172	3ZH2	-10.057	-11.072	4HT4	-11.039	-10.384
4LNQ	-8.011	-9.377	4LJR	-10.244	-9.756	4R56	-9.370	-6.727
4TMU	-12.030	-8.069	4R22	-10.819	-10.377	4Z3C	-7.661	-8.490
3WPC	-10.496	-11.373	3WPD	-11.619	-10.158	2N8A	-9.573	-10.047
5HRT	-12.114	-9.964	5T1J	-10.526	-10.033	5VC9	-9.265	-7.799
5HLG	-8.873	-10.626	5W9Q	-8.482	-7.439	6ASB	-7.940	-9.214
6ASD	-7.661	-8.398	5K07	-6.122	-8.960	5YI2	-9.738	-8.638
6MG3	-11.449	-10.346	6FWR	-8.873	-9.202	6CNP	-8.391	-8.260
5ZD4	-10.798	-9.287	5ZMO	-9.163	-9.864	6BWY	-6.251	-9.403
6CRM	-7.264	-12.078	6CC8	-7.181	-9.082	6BUX	-5.807	-7.718
6A2I	-8.701	-7.413	5ZKI	-8.015	-9.534	6KBS	-7.713	-9.784
5ZKL	-10.479	-10.217	5ZMD	-7.407	-9.116	6ON0	-9.605	-9.809
2A9X	-9.303	-10.954	3BX3	-9.874	-10.191	3IRW	-14.996	-10.855
3IWN	-12.270	-13.425	3K0J	-10.992	-13.428	3QGB	-11.528	-11.217
2XS2	-10.113	-7.906	3QGC	-10.982	-11.066	4GHA	-8.590	-9.489
3V6Y	-11.464	-11.555	2LUP	-6.090	-10.426	4M59	-9.531	-12.065
2MTV	-9.526	-7.928	4RCM	-6.350	-9.135	5EIM	-8.123	-8.359
5GXH	-7.661	-9.283	5KLA	-10.334	-11.348	5EN1	-8.942	-8.923
5F5H	-9.668	-9.273	5WZK	-9.265	-10.222	5HO4	-9.462	-9.199
5U9B	-9.388	-8.892	5WZJ	-10.751	-10.287	5TF6	-11.639	-10.691
5SZE	-10.459	-9.497	5WZG	-10.218	-10.339	5M8I	-5.736	-8.311

PDBID	Exp BA	Pred BA	PDBID	Exp BA	Pred BA	PDBID	Exp BA	Pred BA
5UDZ	-9.592	-9.344	5YTV	-8.043	-7.952	6FQ3	-8.015	-8.838
5YTX	-8.007	-7.982	5YKI	-12.030	-9.948	6DCL	-10.647	-8.916
6GD3	-5.191	-6.825	6G2K	-6.427	-6.511	6CMN	-11.727	-9.292
5YTS	-7.577	-7.671	5WWG	-9.077	-8.855	5YTT	-7.394	-7.775
6FQR	-6.073	-7.321	6GX6	-7.264	-6.222	5WWE	-8.550	-8.906
6GC5	-8.435	-7.224	6RT6	-6.755	-7.762	6NOF	-10.617	-10.431
6R7B	-10.558	-9.540	6NOC	-10.490	-10.469	6NOH	-10.421	-10.387
6A6J	-9.068	-8.487	6NOD	-10.280	-10.526	6G99	-6.176	-9.008
6RT7	-7.405	-7.314	6U9X	-10.086	-9.702	6NY5	-9.952	-10.192
6GBM	-5.556	-9.573	1EC6	-7.990	-9.004	1M8Y	-9.270	-12.497
1RPU	-13.300	-9.771	1UTD	-16.890	-9.030	2B6G	-10.600	-10.012
2ERR	-12.200	-9.110	2F8K	-10.300	-9.927	2G4B	-7.830	-8.468
2KFY	-8.840	-8.126	2KG0	-7.400	-8.128	2KG1	-7.450	-8.131
2KX5	-11.400	-9.967	2L41	-4.250	-7.744	2LA5	-11.500	-10.760
2LEB	-9.320	-8.734	2LEC	-9.440	-8.680	2M8D	-8.730	-8.680
2MJH	-9.670	-8.771	2MXY	-7.900	-6.914	2MZ1	-6.870	-7.871
2N82	-10.300	-10.363	2RU3	-8.520	-9.081	2XC7	-7.040	-8.859
2XFM	-8.240	-7.836	2XNR	-5.400	-7.462	2ZKO	-8.050	-10.219
3BSB	-10.200	-10.955	3BSX	-11.500	-10.696	3BX2	-9.970	-9.730
3K49	-12.050	-10.952	3K4E	-10.800	-11.859	3K5Q	-9.300	-9.072
3K5Y	-10.540	-9.511	3K5Z	-9.380	-9.682	3K61	-8.890	-9.531
3K62	-8.740	-9.253	3K64	-9.220	-8.886	3L25	-8.175	-9.260
3LQX	-12.220	-10.909	3MDG	-7.850	-8.917	3MOJ	-13.000	-10.957
3NCU	-10.300	-9.814	3NMR	-8.440	-7.299	3NNH	-6.190	-8.698
3O3I	-6.520	-7.070	3O6E	-7.070	-6.524	3Q0L	-12.300	-11.392
3Q0M	-12.500	-12.818	3Q0N	-10.500	-10.804	3Q0P	-12.800	-10.043
3Q0Q	-13.300	-12.542	3Q0R	-13.800	-12.954	3Q0S	-10.800	-11.713
3QG9	-10.600	-11.263	3U4M	-15.900	-10.085	3V71	-11.100	-10.371
3V74	-11.600	-11.382	3WBM	-9.980	-10.822	4CIO	-9.760	-9.002
4ED5	-9.080	-8.516	4ERD	-9.540	-10.329	4JVH	-9.590	-9.484
4KJI	-8.940	-10.146	4LG2	-9.540	-8.843	4NL3	-7.380	-10.077
4O26	-8.200	-11.145	4OE1	-12.110	-9.541	4QI2	-9.440	-9.828
4QVC	-11.560	-9.994	4QVD	-10.120	-11.377	4R3I	-7.770	-6.765
4RCJ	-8.180	-8.440	4TUX	-10.400	-9.287	4U8T	-9.130	-7.841
4Z31	-8.790	-9.306	5DNO	-7.830	-8.538	5ELR	-6.250	-8.793
5V7C	-6.990	-9.070	5W1I	-12.790	-10.243	5WZH	-10.460	-10.308
6D12	-9.300	-10.271						

References

[1] Li Shen, Hongsong Feng, Yuchi Qiu, and Guo-Wei Wei. Svsbi: sequence-based virtual screening of biomolecular interactions. *Communications biology*, 6(1):536, 2023.
